# Delay eyeblink conditioning performance and brain-wide c-Fos expression in male and female mice

**DOI:** 10.1098/rsob.220121

**Published:** 2023-05-10

**Authors:** Maria Roa Oyaga, Ines Serra, Devika Kurup, Sebastiaan K. E. Koekkoek, Aleksandra Badura

**Affiliations:** ^1^ Department of Neuroscience, Erasmus MC, 3000 Rotterdam, The Netherlands; ^2^ Netherlands Institute of Neuroscience, Royal Dutch Academy for Arts and Sciences, Amsterdam 1105 BA, The Netherlands

**Keywords:** eyeblink, learning, locomotion, c-Fos, sex, strain

## Abstract

Delay eyeblink conditioning has been extensively used to study associative learning and the cerebellar circuits underlying this task have been largely identified. However, there is a little knowledge on how factors such as strain, sex and innate behaviour influence performance during this type of learning. In this study, we used male and female mice of C57BL/6J (B6) and B6CBAF1 strains to investigate the effect of sex, strain and locomotion in delay eyeblink conditioning. We performed a short and a long delay eyeblink conditioning paradigm and used a c-Fos immunostaining approach to explore the involvement of different brain areas in this task. We found that both B6 and B6CBAF1 females reach higher learning scores compared to males in the initial stages of learning. This sex-dependent difference was no longer present as the learning progressed. Moreover, we found a strong positive correlation between learning scores and voluntary locomotion irrespective of the training duration. c-Fos immunostainings after the short paradigm showed positive correlations between c-Fos expression and learning scores in the cerebellar cortex and brainstem, as well as previously unreported areas. By contrast, after the long paradigm, c-Fos expression was only significantly elevated in the brainstem. Taken together, we show that differences in voluntary locomotion and activity across brain areas correlate with performance in delay eyeblink conditioning across strains and sexes.

## Graphical abstract

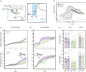

## 1. Introduction

Eyeblink conditioning is a behavioural paradigm commonly used to study cerebellar-dependent associative learning. It has been applied in a variety of animals including mice, cats, rats, rabbits and ferrets [1–5], and has also been used in human research to identify behavioural deficits in Parkinson's disease, multiple sclerosis and autism spectrum disorder. [6–8]. In this task, an initially neutral, conditioned stimulus (CS; a flashing light or sound), becomes predictive of an unconditioned stimulus (US; an air puff to the cornea), which elicits a blink of the eye. The paradigm consists of pairing the CS with the US and, over time, an association is formed where blinking is triggered by the presentation of the CS alone. The newly learned association is called conditioned response (CR) [9]. The most commonly used version of this task is the so-called delay eyeblink conditioning paradigm, in which the presentation of the US immediately follows the CS with both of the stimuli ending at the same time [10].

At the circuit level, studies have shown that the association between stimuli during delay eyeblink conditioning relies on the cerebellum. Here, the CS signals coming from the pons and the US information (puff) from the inferior olive via climbing fibres are precisely timed and processed [11,12]. Consequently, several cerebellar areas modulating eyeblink conditioning have been identified in mice, including lobule VI in the vermal region, and crus 1 and simplex in the hemispheric region [13,14]. Inactivation of lobule VI and crus 1 during development causes deficits in learning, supporting their crucial role in eyeblink conditioning [15]. The CR signal leaves the cerebellum via the interposed nucleus, which ultimately connects to the muscles controlling the eyeblink reflex [2,14]. Beyond the ponto-cerebellar and olivo-cerebellar systems, little is known about the potential involvement of other brain areas in delay eyeblink conditioning [16,17], although the amygdala has also been proposed to have a role in this form of associative learning, given its implication in fear conditioning and arousal [18].

Despite extensive knowledge on the cerebellar substrates involved in eyeblink conditioning, our understanding of how intrinsic factors, such as sex, strain and behaviour, contribute to the variability in performance during this task remains limited. As with the vast majority of behavioural mouse tasks, male C57BL/6 (B6) mice are preferentially used to investigate eyeblink performance, with other strains and female mice remaining considerably under-investigated. While strain limitations are usually associated with the use of inbred strains with intention to decrease variability and increase statistical power [19], the underuse of females relates to the presumption that hormonal fluctuations caused by the estrous cycle introduce non-comparable variability across sexes [20]. This remains a common belief despite meta-analyses of rat and mice studies showing that females and males exhibit comparable behavioural, morphological and physiological trait variability [21,22]. In general, knowledge on the contribution of sex towards performance variability in delay eyeblink conditioning is scarce and often conflicting. In humans, females produce more CRs than males both in children below the age of nine, and in adults [23]. In rats, stress enhances conditioning responses but hinders learning in females [24]. However, in rabbits, both males and females show similar conditioning profiles, with females adapting faster to stress [25]. A recent study of trace eyeblink conditioning, a paradigm in which the US is separated from the CS by a time interval, showed that both sexes reached similar learning scores but female mice showed significantly higher CR percentage compared to males in the first 5 days of learning [26]. Given this heterogeneity of results, gaining a deeper understanding of the effects of sex in eyeblink conditioning performance is crucial to better understand which parameters influence learning during this task.

Sex and strain are not the only variables that can potentially influence mice performance during delay eyeblink conditioning. This task is most commonly performed in head-fixed mice freely running on a wheel and studies have shown that locomotion speed on the wheel positively correlates with a higher percentage of CRs [27,28]. However, it is still unknown if locomotion strategy during delay eyeblink conditioning is sex dependent. This is highly plausible, given that sex-specific strategies in mice were previously observed in locomotion adaptation [29], exercise capacity [30] and spatial orientation and learning [31], where female mice learned faster.

Of note, brain activity in a number of areas involved in delay eyeblink conditioning is modulated by locomotor activity. In the cerebellum, locomotion changes the baseline neuronal activity in the cortex [32,33], although the relevance of this modulation towards associative learning is still not fully understood. Arousal and locomotion influence spontaneous and evoked activity in the neocortex and thalamus [34,35], and recruit many brainstem regions [36]. How locomotion modulates the activation of distinct brain areas during delay eyeblink conditioning, particularly outside of the cerebellar circuit, remains an open question. One method to evaluate brain activity in areas involved in a learning task is through c-Fos detection. *c-fos* is an immediate early gene, whose expression in neurons is relatively low at rest, but rapidly increases shortly after depolarization, especially following learning paradigms [37]. Upon neuronal activation, *c-fos* mRNA reaches a maximum expression level after approximately 30 min, while protein expression peaks within 1 to 2 h post-activation [38]. Because of this precise time window of expression, and its increase after exposure to novel objects, surroundings or stimuli, c-Fos is widely used as a neuronal activity marker [39–41]. In mice, it has been commonly used to investigate seizures [42–44], contextual conditioning and memory [45,46] and social behaviours [47–50]. c-Fos immunohistochemistry has several advantages: it is a non-invasive technique that allows quantification of brain wide neural activity, it detects activation in deep nuclei that would be otherwise hard to reach with electrophysiology, it is fast and relatively easy to perform, it allows cellular resolution, and it can be used in combination with other quantitative techniques, for example, with other cell-type specific immunostainings [51,52]. However, it also has its limitations: it depicts transcriptional activation of neurons that are activated, not inhibited, hence, it is limited to the study of excitatory events and its temporal resolution is low. Finally, the presence of c-Fos expression does not provide any information about the connectivity patterns of the areas where the expression can be visualized [51,52]. How the c-Fos expression correlates with learning in different sexes and strains, and across distinct areas following delay eyeblink conditioning remains elusive.

Here, using male and female mice of the B6 and B6CBAF1 backgrounds, we investigated the effects of sex, strain and spontaneous locomotion in delay eyeblink conditioning performance. Furthermore, by using c-Fos expression as a proxy for neural activity during learning, we explored how sex, strain and locomotion activity modulate the engagement of distinct brain regions during delay eyeblink conditioning. Using a short 5-day and a standard, long 10-day paradigm, we found that B6 male and female mice showed comparable variability in the delay eyeblink conditioning with females outperforming males at day 5. This sex difference in the performance was also found in the B6CBAF1 mice exposed to the 5-day paradigm, but is no longer present in B6 mice exposed to 10 days of training. Further, we found a strong positive correlation across sexes between learning scores and voluntary locomotion in the B6 mice. Following the short training protocol, c-Fos immunostaining revealed positive correlations between c-Fos positive cell density and learning scores in the cerebellar cortex, as well as multiple previously unreported extra-cerebellar areas. Examination of the c-Fos signal in mice subjected to the long paradigm showed a significant correlation with learning scores solely in the brainstem. These results are congruous with previous studies, which show that cerebellar cortex is necessary for the acquisition of the CRs but not the execution of the learned responses [16,53–55].

## 2. Results

### 2.1. B6 female and male mice show comparable variability in delay eyeblink conditioning but female learning scores peak faster

**Figure 1.**
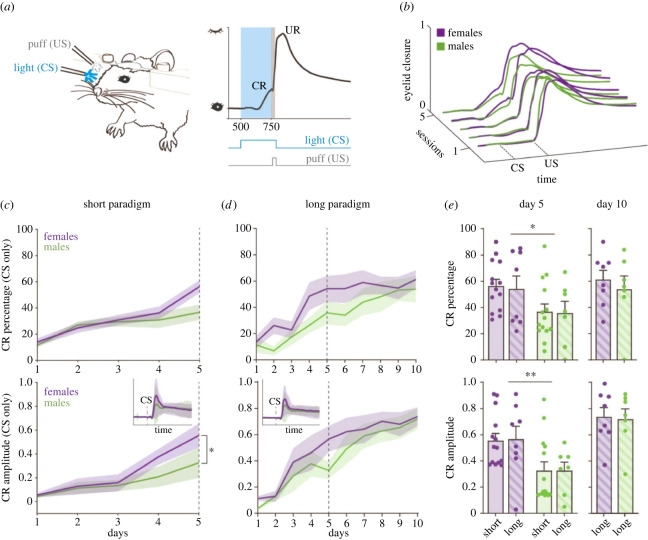
B6 female and male mice show comparable variability in eyeblink conditioning and females reach higher learning scores faster. (*a*) Experimental set-up. Mouse with an implanted headplate is head-fixed on top of a freely rotating wheel. A blue light (CS) is presented 250 ms before a puff (US) to the same eye. In a trained mouse, the CS produces an anticipatory eyelid closure (CR), followed by a blink reflex triggered by the US (UR). (*b*) Paired trials average traces in females and males during the short (5-day) paradigm. The CR progressively develops due to the CS–US pairing. (*c,d*) CR percentage and amplitude in CS-only trials over the short (*c*) and long (10-day) (*d*) paradigm. Inset shows average response in CS-only trials on day 5 of training. Purple, females; green, males; shaded area, s.e.m. (*e*) Mean percentage and amplitude response in CS-only trials, comparing the performance of the cohorts used in the short and long paradigms. Differences between female and male performance are no longer detected on day 10. Purple, females (*n* = 14 short, *n* = 8 long); green, males (*n* = 14 short, *n* = 7 long); *p < 0.05, **p < 0.01.

To study differences in learning profiles between sexes, we performed delay eyeblink conditioning experiments with B6 females and males. First, we habituated the animals to the set-up for increasing periods of time over 5 days to decrease anxiety levels and optimize mouse position on the running wheel. Next, we subjected separate batches of mice to a short or long (5- or 10-day) training paradigm in order to capture behavioural variability in distinct stages of learning. The blue LED light (CS) was triggered 250 ms prior to the puff to the cornea (US) in paired trials and the two stimuli co-terminated (figure 1*a*). Daily sessions consisted of 20 blocks of 12 trails each (1 US-only, 11 paired and 1 CS-only). Mice learned the association between the stimuli progressively and developed a CR that gradually increased over time (figure 1*b*).

In the short, 5-day paradigm, females showed an increase in CR percentage in CS-only trials on session four that culminated with a 60% CR responses in session five opposed to 40% in males (two-way ANOVA repeated measures for sex and session × sex effect: *F*_1,26_ = 1.461, *p* = 0.24, interaction sex and session: *F*_4,104_ = 4.01, *p* = 0.02, Cohen's *d* session five: 0.88) (figure 1*c*, top panel). The CR amplitude was normalized to maximum eye closure during the US-only trials, which triggered an unconditioned response (UR, i.e. blink). The UR maximum amplitude (i.e. peak amplitude = 1) was therefore defined as the maximal eye closure in response to the puff. The CR responses were normalized for each mouse, within each session as daily adjustments of the mouse position on the wheel translated to small variations in the eye position across sessions (see Material and methods). The CR amplitude in CS trials was significantly higher in females compared to males (two-way ANOVA repeated measures for sex and session × sex effect: *F*_1,26_ = 6.109, *p* = 0.02, interaction sex and session: *F*_4,104_ = 5.12, *p* = 0.0008, Cohen's *d* session five: 0.93) (figure 1*c*, bottom panel). On day 5, females reached average amplitude of 0.55 while males reached an average of 0.33 (figure 1*c*, inset).

Next, we performed a standard, 10-day training paradigm in a second cohort of mice (figure 1*d*). Congruous with the results from the first cohort, females showed higher CR percentage and amplitude after 5 days of training (two-way ANOVA; effect of paradigm on percentage: *F*_1,39_ = 0.051, *p* = 0.82; effect of paradigm on amplitude: *F*_1,39_ = 0.01, *p* = 0.94) (figure 1*e*). However, at the end of the long paradigm, these sex differences were no longer significant (two-way repeated measures ANOVA; effect of sex on percentage: *F*_1,13_ = 2.10, *p* = 0.17; effect of sex on amplitude: *F*_1,13_ = 0.97, *p* = 0.34) (figure 1*e*). Overall, these results show that male and female mice display comparable variance in delay eyeblink conditioning and that females acquire higher learning scores faster, although these sex differences dissipate after 10 days of training.

### 2.2. Learning scores correlate with spontaneous locomotor activity

We next asked whether mice showed sex-dependent behavioural differences in voluntary locomotor activity during training, as it has been previously shown that locomotion enhances learning in delay eyeblink conditioning [27]. For this purpose, we added infrared cameras to the eyeblink set-ups to record body movement during eyeblink sessions. The cameras were placed at the right back corner of the box, allowing a wide recording angle to capture whole-body movements (electronic supplementary material, figure S1A). We recorded videos of full training sessions for each mouse, which were later analysed offline. The head bar height (figure 1*a*) was adjusted accordingly to ensure that every mouse could move comfortably on the wheel. To track different body parts and get a meaningful movement output, we used DeepLabCut (DLC), a software for automated animal pose tracking [56] (see Material and methods, Locomotion analysis). This approach allows movement tracking without using physical markers on the body that can hinder natural movement. We tracked five body parts: tail base, hip, knee, right back paw and nose (electronic supplementary material, figure S1A). Animals were head-fixed on top of the wheel; hence, *y* position was similar between both sexes (electronic supplementary material, figure S1B,C). We selected the speed of the right back paw as a proxy for general locomotion behaviour for further analysis. We also observed more fluctuations in the hip and tail position along the *x*-axis in males (electronic supplementary material, figure S1B,C).

**Figure 2.**
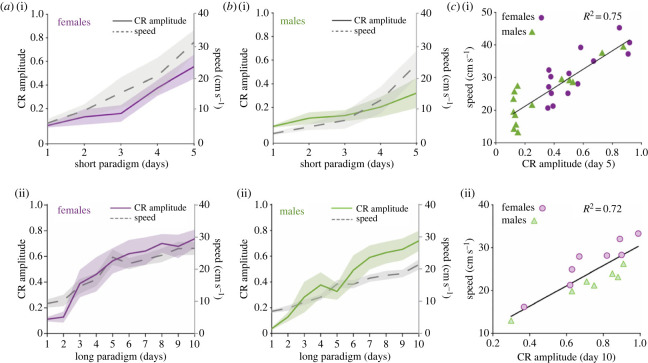
Learning scores correlate with spontaneous locomotor activity. (*a,b*) CR amplitude and speed of the right back paw over training sessions for the short (i) and long (ii) training paradigms. Purple, females (*n* = 14 short, *n* = 8 long); green, males (*n* = 14 short, *n* = 7 long); shaded area, s.e.m. Speed: two-way ANOVA for sex and session × sex effect short paradigm: *F*_1,26_ = 12.17, *p* = 0.0017; sex effect long paradigm *F*_1,13_ = 7.81, *p* = 0.02. (*c*) Positive correlation between CR amplitude and speed of the right back paw on the last session of training (linear regression: short paradigm *R*^2^ = 0.75, *p* = 0.002; long paradigm *R*^2^ = 0.72, *p* < 0.0001).

Animals increased their speed on the wheel during short and long training paradigms, and both females and males had comparable variances (*F*-test for two-sample variances in speed: *F* = 0.58, *p* = 0.305). We found that females moved significantly faster than males during learning, reaching on the last day an average speed of 31 and 27 cm s^−1^ compared to males that reached 24 and 21 cm s^−1^ during the short and long paradigms, respectively (two-way ANOVA repeated measures for sex and session × sex effect in short paradigm: *F*_1,26_ = 12.17, *p* = 0.0017, Cohen's *d* session five: 1.07; effect of sex in long paradigm: *F*_1,13_ = 7.81, *p* = 0.02) (figure 2*a,b*). Because we observed a similar trend between CR amplitude and running speed across sexes and paradigms, we performed a linear regression between speed of the right back paw and CR amplitude in the last session of each paradigm. This showed a clear correlation between the variables (short paradigm: *R*^2^ = 0.75, *p* = 0.002; long paradigm: *R*^2^ = 0.72, *p <* 0.0001) (figure 2*c*). These results reveal that mice that spontaneously move faster on the wheel, reach higher learning scores in delay eyeblink conditioning.

### 2.3. Learning scores correlate with c-Fos expression

To explore if differences in eyeblink performance correlate with differences in brain activity, we performed c-Fos immunostainings after the last training session of the short and long paradigm. We imaged sections of whole brains with a fluorescent microscope and developed an image analysis workflow to quantify c-Fos positive neurons and identify their location within the hierarchical structure of the Allen Brian Atlas (cerebrum, brainstem and cerebellum) (electronic supplementary material, figure S2). To identify regions potentially involved in associative learning, we first selected mice that showed a CR amplitude of 0.4 or higher in CS-only trials (*n* = 14, 5 B6 males and 9 B6 females for the short paradigm; *n* = 13, 6 B6 males and 7 B6 females for the long paradigm). These mice are further referred to as ‘high learners’. We performed Kendall's correlation between the density of c-Fos positive cells and the CR amplitude reached during the last session.

**Figure 3.**
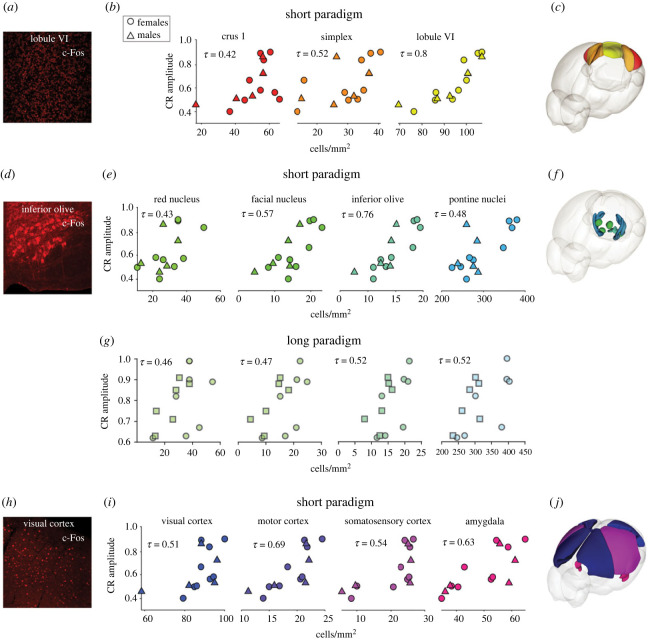
Learning scores correlate with c-Fos expression. (*a*) c-Fos positive granule cells in lobule VI in the cerebellum. (*b*) Cerebellar areas with a significant positive correlation between c-Fos positive cell density and CR amplitude (crus 1: tau = 0.42, *p* = 0.042, simplex: tau = 0.52, *p* = 0.009, lobule VI: tau = 0.8, *p* = 0.009). (*c*) Three-dimensional model with significant areas highlighted. (*d*) c-Fos positive cells in the inferior olive. (*e*) Brainstem areas with a significant positive correlation between c-Fos positive cell density and CR amplitude (red nucleus: tau = 0.43, *p* = 0.041, facial nucleus: tau = 0.57, *p* = 0.006, inferior olive: tau = 0.76, *p* = 0.0008, pontine nuclei: tau = 0.48, *p* = 0.021). (*f*) Three-dimensional model with significant areas highlighted. (*g*) Brainstem areas with a significant positive correlation between c-Fos positive cell density and CR amplitude in the long paradigm (red nucleus: tau = 0.46, *p* = 0.04), facial nucleus: tau = 0.47, *p* = 0.03, inferior olive: tau = 0.52, *p* = 0.02, pontine nuclei: tau = 0.52, *p* = 0.02). (*h*) c-Fos positive cells in the visual cortex. (*i*) Cortical areas with a significant positive correlation between c-Fos positive cell density and CR amplitude (visual cortex: tau = 0.51, *p* = 0.013, motor cortex: tau = 0.69, *p* = 0.0003, somatosensory cortex: 0.54, *p* = 0.007, amygdala: tau = 0.63, *p*: 0.001). (*j*) Three-dimensional model with significant areas highlighted.

In the cerebellum, c-Fos labelling was localized in the granule cell layer in the mice that underwent the short paradigm training, which we confirmed with colocalization with GABA*α*6, a granule cell-specific marker (figure 3*a*; electronic supplementary material, figures S3 and S4A,B). In the cerebellar hemispheric regions, crus 1 and simplex had a significant correlation between c-Fos cell density and CR amplitude (crus 1: tau = 0.42, *p* = 0.042, simplex: tau = 0.52, *p* = 0.009). In the cerebellar vermis, lobule VI also had a significant correlation and the highest Tau (lobule VI: tau = 0.8, *p* = 0.009) (figure 3*b,c*). No significant correlations were found between c-Fos expression and CR amplitude in cerebral or cerebellar areas following the 10-day training paradigm (electronic supplementary material, table S1).

In the brain stem, we found a significant correlation in the red nucleus, the facial nucleus, the inferior olive and the pontine nuclei in the short and long paradigm cohorts (short paradigm: red nucleus: tau = 0.43, *p* = 0.041, facial nucleus: tau = 0.57, *p* = 0.006, inferior olive: tau = 0.76, *p* = 0.0008, pontine nuclei: tau = 0.48, *p* = 0.021; long paradigm: red nucleus tau = 0.46, *p* = 0.04, facial nucleus: tau = 0.47, *p* = 0.03, inferior olive: tau = 0.52, *p* = 0.02, and pontine nuclei: tau = 0.52, *p* = 0.02; figure 3*d–g*; electronic supplementary material, figure S3 and table S1).

Significant positive correlations in the visual, motor and somatosensory cortices, and the amygdala were detected only in mice that underwent the short training (visual cortex: tau = 0.51, *p* = 0.013, motor cortex: tau = 0.69, *p* = 0.0003, somatosensory cortex: 0.54, *p* = 0.007, amygdala: tau = 0.63, *p =* 0.001) (figure 3*h–j*; electronic supplementary material, figure S3 and table S1). Double immunostainings using c-Fos and CUX1, a marker for upper cortical layers (II–IV), or CTIP2, for lower cortical layers (V–VI), revealed c-Fos positive cells in all layers of the cortex following the short training paradigm (electronic supplementary material, figure S4C–F).

Together, these results confirm the involvement of previously reported areas associated with delay eyeblink conditioning within the olivo-cerebellar and ponto-cerebellar systems [17,57] and suggest that other areas might be involved in the acquisition of this learning task. Furthermore, the results of the long training are in line with the previous reports that suggest transfer of learned responses away from cerebellar cortex [16,53–55] and neocortex once the learning is consolidated [58].

### 2.4. Pseudoconditioned mice show lower c-Fos expression

To ensure an appropriate control for the quantification of c-Fos positive cells, we included pseudoconditioned mice (*n* = 8 for the short paradigm, 4 B6 males and 4 B6 females; *n* = 3 for the long paradigm, 2 B6 males and 1 B6 female) in our experimental design. These mice went through the same experimental steps as the conditioned mice, with the only exception that they were not trained with paired CS-US trials. Instead, we exposed them to a protocol with CS- and US-only trials, keeping the same structure and duration as the conditioned protocol and tracking their locomotion behaviour throughout the experiment.

**Figure 4.**
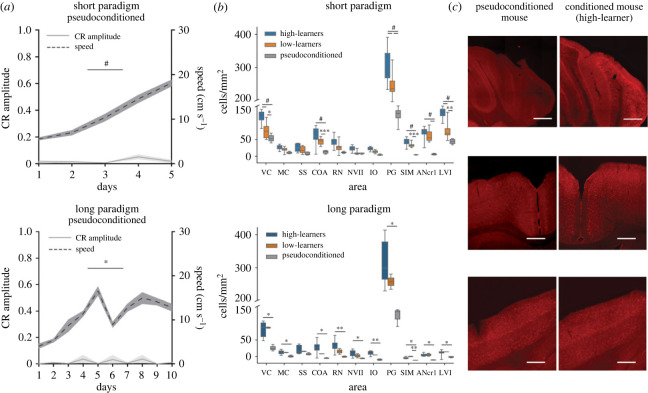
Pseudoconditioned mice show lower c-Fos expression compared to high and low learners. (*a*) Change in CR amplitude and speed of the right back paw of pseudoconditioned mice over training sessions for the short (top; *n* = 8) and long (bottom; *n* = 3) paradigms. CR amplitude in the left *y*-axis and speed in the right *y*-axis. (*b*) Boxplots depict c-Fos density in high learners (CR amplitude > 0.4 on last session; *n* = 14 short paradigm, *n* = 13 long paradigm), low learners (CR amplitude < 0.4 on last session; *n* = 14 short paradigm, *n* = 2 long paradigm) and pseudoconditioned mice on the short (top) and long (bottom) paradigm. (*c*) Representative immunofluorescent images of c-Fos positive cells in a pseudocontidoned mouse (left) and a high-learner mouse (right). Top to bottom: cerebellar cortex, motor cortex, somatosensory cortex. Scale bar: 100 μm. *p < 0.05, **p < 0.01, ***p < 0.001, ^#^*p* < 0.0001.

Pseudoconditioned mice did not acquire an association given that there was no substrate for learning (absence of CS-US pairing) but, similar to the conditioned mice, did increase the locomotion speed over training sessions (short paradigm: *F*_2,733, 19,13_ = 49.23, *p* < 0.0001; long paradigm: *F*_1,749, 3,497_ = 19.28, *p* = 0.01) (figure 4*a*). We applied the same analysis method to quantify the density of c-Fos positive cells in pseudoconditioned mice as described above. Overall, we observed lower c-Fos expression in pseudoconditioned mice compared to high learners and low learners (effect of learning: short paradigm- *F*_2,363_ = 251.5, *p* < 0.0001; mean c-Fos density high learners = 61.74, low learners = 44.45, pseudoconditioned = 17.83; long paradigm H(2) = 28.41, *p* < 0.001; median c-Fos density high learners = 18.46, low learners = 15.84, pseudoconditioned = 6.77) (figure 4*b*; electronic supplementary material, figure S5 and table S2). We then compared the c-Fos density between pseudoconditioned, high and low learners. We specifically focused on the brain areas where we found a significant correlation between CR amplitude and c-Fos density during the short paradigm (figure 3). In the short paradigm, both high and low learners had significantly higher c-Fos density mean compared to pseudoconditioned mice in the VC, COA, PG, SIM, ANcr1 and LVI (figure 4*b* top panel; figure 4*c*). Differences between high and low learners were also observed in the VC, PG and LVI. Following the long paradigm, no differences in c-Fos density were found between high and low learners (figure 4*b* bottom panel), while increased c-Fos density was found across all areas (except for SS) between high-learners and pseudoconditioned mice. This suggests that the observed increased c-Fos expression in a number of brain areas is related to the learning of the CS-US pairing and not just to the locomotion or sensory stimulation (light and puff) alone.

### 2.5. Correlation between learning scores and c-Fos expression is consistent in B6CBAF1 strain

Given the evidence of the possible unwanted effects of highly inbred mouse strains like B6 in replicability and reproducibility [59], we wanted to investigate inter-strain variability in associative learning. We asked whether the results obtained in B6 mice could be observed in a different mouse strain. For this purpose, we subjected B6CBAF1 mice (the F1 hybrids of B6 and CBA strains) to the short delay eyeblink conditioning paradigm. B6CBAF1 mice have significantly less retinal degeneration and hearing loss compared to B6 mice, which makes them favourable models for visual and auditory experiments [60–62].

**Figure 5.**
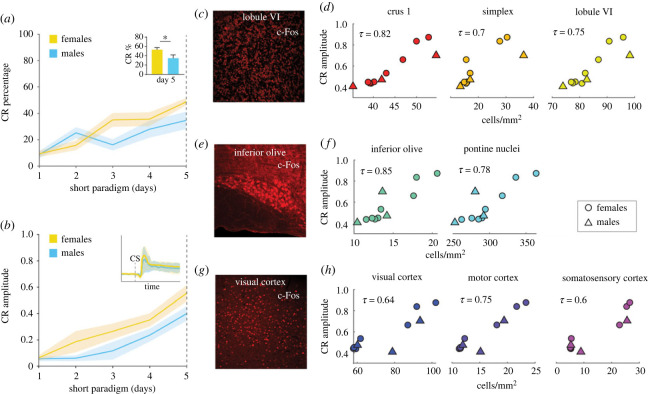
Correlation between learning scores and c-Fos expression is consistent in B6CBAF1 mice. (*a*) CR percentage in CS only trials over training sessions. Yellow, females (*n* = 9); cyan, males (*n* = 7); shaded area, s.e.m. (*b*) CR amplitude in CS only trials over training sessions. Shaded area, s.e.m. (*c*) c-Fos positive cells in lobule VI. (*d*) Cerebellar areas with a significant positive correlation between c-Fos positive cell density and CR amplitude in high learners (crus 1: tau = 0.82, *p* = 0.0001, simplex: tau = 0.7, *p* = 0.005, lobule VI: tau = 0.75, *p =* 0.0007). (*e*) c-Fos positive cells in the inferior olive. (*f*) Brainstem areas with a significant positive correlation between c-Fos positive cell density and CR amplitude in high learners (inferior olive: tau = 0.85, *p =* 0.0004, pontine nuclei: tau = 0.78, *p =* 0.0003). (*g*) c-Fos positive cells in the visual cortex. (*h*) Cortical areas with a significant positive correlation between c-Fos positive cell density and CR amplitude in high learners (visual cortex: tau = 0.64, *p* = 0.0057, motor cortex: tau = 0.75, *p* = 0.0008, somatosensory cortex: tau = 0.6, *p =* 0.009).

B6CBAF1 mice learned the association between the stimuli and gradually formed CRs. We observed a trend indicating similar sex differences between B6CBAF1 mice and B6. Females reached 53% CR percentage compared to 35% in males (two-way ANOVA repeated measures for sex and session × sex effect: *F*_1,14_ = 2.55, *p* = 0.237, interaction sex and session: *F*_4,104_ = 3.01, *p* = 0.021, Cohen's *d* session five: 0.86) (figure 5*a*). When it comes to the amplitude of these responses, BFCBAF1 females showed a trend towards slightly higher CR amplitude over training sessions compared to males (two-way ANOVA repeated measures for sex and session × sex effect: *F*_1,14_ = 2.55, *p* = 0.132, Cohen's *d* session five: 0.84) (figure 5*b*).

Following the last day of learning, the B6CBAF1 mice were perfused and we quantified the whole-brain c-Fos expression (electronic supplementary material, figure S6). We selected mice that showed a CR amplitude of 0.4 or higher in CS-only trials (*n* = 11; 3 males and 8 females) and performed Kendall's correlation between density of c-Fos positive cells and CR amplitude on the last training session. The granule cell layer also contained c-Fos labelling, and crus 1, the simplex and lobule VI were found to have a significant positive correlation (crus 1: tau = 0.82, *p* = 0.0001, simplex: tau = 0.7, *p* = 0.005, lobule VI: tau = 0.75, *p* = 0.0007) (figure 5*c,d*; electronic supplementary material, figure S7). In the hindbrain, the correlation between c-Fos cells and learning was also significant in the inferior olive and the pontine nuclei (inferior olive: tau = 0.85, *p* = 0.0004, pontine nuclei: tau = 0.78, *p* = 0.0003) (figure 5*e*,*f*; electronic supplementary material, figure S7). Additionally, the visual, motor and somatosensory cortices showed significant positive correlations (visual cortex: tau = 0.64, *p* = 0.0057, motor cortex: tau = 0.75 *p* = 0.0008, somatosensory cortex: tau = 0.6, *p* = 0.009) (figure 5*g*,*h*; electronic supplementary material, figure S7).

These results show that most of the areas where we saw a correlation between learning and c-Fos density in B6 mice are also found in B6CBAF1 mice, which strengthens the idea that these areas are active during delay eyeblink conditioning.

### 2.6. Variability between sexes and strains

**Figure 6.**
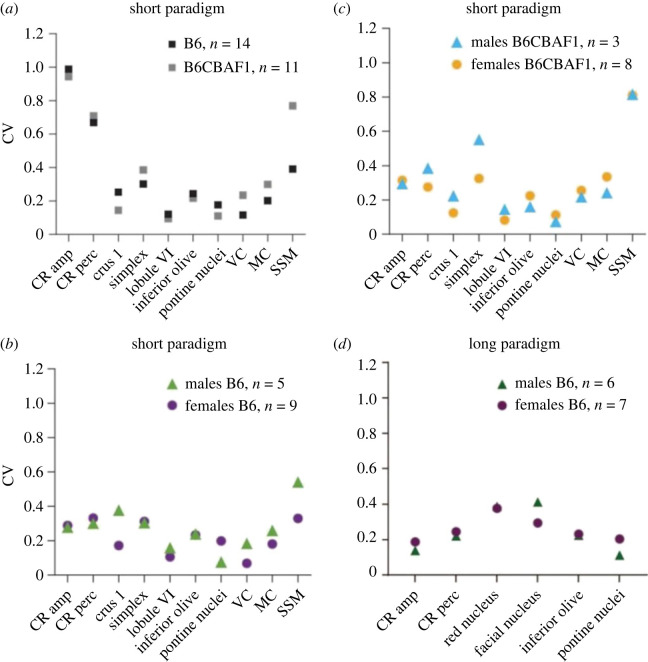
Strain and sex variability. High learners were selected based on CR amplitude on the last training session > 0.4. CV = s.d./mean. (*a*) For strains: B6, *n* = 14, B6CBAF1, *n* = 11. (*b*) For B6 mice: males, *n* = 5; females, *n* = 9. (*c*) For B6CBAF1 mice: males, *n* = 3; females, *n* = 8. (*d*) For the B6 long paradigm: males, *n* = 6; females, *n* = 7. VC, visual cortex; MC, motor cortex; SSM, somatosensory cortex; CV, coefficient of variation.

To further understand inter-strain and inter-sex variability in our dataset, we calculated the coefficient of variance (CV, s.d./mean) for each of the variables that we quantified in both B6 and B6CBAF1 mice. For mice that were subjected to the short paradigm, we selected the 14 high learners (CR amplitude on session 5 > 0.4) B6 mice (*n* = 5 males, 9 females) and the 11 high-learners B6CBAF1 mice (*n* = 3 males, 8 females) and grouped them by strain and sex (figure 6). For each group, we calculated the CV for the common variables acquired and previously reported, which can be grouped in two main categories: eyeblink performance and c-Fos expression. Eyeblink performance includes CR amplitude and percentage on the last day of training. c-Fos expression includes the density of c-Fos positive cells in the brain areas where we have found a positive significant correlation across both strains, during the short paradigm: crus 1, simplex, lobule VI, inferior olive, pontine nuclei, and the visual, motor and somatosensory cortices. When comparing strains, we observed that the variances for each variable were similar, with the exception of the density of c-Fos positive cells in the somatosensory cortex, where B6CBAF1 mice seem to be more variable compared to B6 (figure 6*a*). B6 female and male mice had similar CVs for most of the variables, although in crus 1 and the somatosensory cortex males seem to have a slightly higher CV (figure 6*b*). However, this could be due to the difference in sample size. We observed something similar between B6CBAF1 female and male mice; males showed slightly higher CV in c-Fos density in the simplex (figure 6*c*). Additionally, B6CBAF1 mice had the highest CV in the somatosensory cortex. When we calculated the CR amplitude variance across all days of training in the short paradigm, female mice presented with higher variance compared to male mice (*F*-test for two sample variances in CR amplitude of paired trials- B6: *F* = 1.74, *p* = 0.01; B6CBAF1: *F* = 1.77, *p* = 0.04), reflecting the higher number of high learners in the female population in the early stages of training. Notably, both B6 and B6CBAF1 females and males presented with very similar variances (B6 females = 0.052, B6CBAF1 females = 0.058; B6 males = 0.030, B6CBAF1 males = 0.033). In the long paradigm cohort, we analysed CVs for the eyeblink performance variables, as well as for the brain areas where we have found a positive significant correlation: red nucleus, facial nucleus, inferior olive and pontine nuclei (figure 3*g*). Compared to the short paradigm data, long paradigm B6 males and B6 females present with increasing overlapping CVs in the analysed variables (figure 6*d*). Furthermore, the analysis of CR amplitude variance across the 10 days of training showed no effect of sex on this variable (*F* = 1.15, *p* = 0.28), suggesting that both eyeblink performance and c-Fos expression become more similar between sexes with increasing training time.

## 3. Discussion

Understanding behavioural variability in the context of neuroscience research is a challenge. We are still far from fully comprehending how factors like sex and strain, and differences in baseline activity levels give rise to differences in performance in learning tasks. Here we addressed this question by making use of a well-known learning paradigm to study behavioural variability. We found that B6 and B6CBAF1 mice showed comparable variance in delay eyeblink conditioning and locomotion while being head-fixed on a rotating wheel. The differences between sexes were evident with females reaching higher learning scores and running speeds within 5 days of training, but dissipated after a 10-day paradigm. Importantly, we found a robust correlation between learning scores and running speed, which was consistent across sexes and strains. In a similar way, we found that enriched c-Fos expression across several brain areas positively correlates with learning, which suggests the involvement of these regions in eyeblink conditioning.

### 3.1. Sex, strain and behaviour: comparable variability but difference in performance

Opposed to what is sometimes assumed in behavioural studies, we observed that sexes show comparable variability as the training progressed. However, we found sex-dependent differences in performance during the early stages of the acquisition of delay eyeblink conditioning, and in the locomotion patterns.

Past studies on sex differences in rodents showed that many behaviours vary between females and males [63]. This is true for innate behaviours such as running [64,65], where female mice show increased duration and speeds of locomotion compared to males, as well as in learned responses measured in laboratory settings, such as spatial learning [66], where male rats outperform females. However, data on sex differences in research is often conflicting, with several studies not finding sex-dependent behavioural differences [67,68]. These divergent results can be attributed to a variety of causes. However, increasing studies point to differences in behavioural strategies and differential expression of fear behaviour as sources for result variability [69,70]. Behavioural sex differences have also been studied in multiple other species. In addition to differences in mating behaviours, non-reproductive behavioural differences have also been found in fish, including increased discrimination in reversal learning in females [71,72]. In rhesus monkeys, females perform better in a spatial delayed-response task [73], while young female chimpanzees exhibit thermite fishing behaviour earlier than males [74]. Notably, conflicting reports on sex-divergent behaviours can also be found in humans, with some studies pointing to significant sex effects [75–77], where others fail to show any significant differences between sexes [78]. Therefore, generalizations of sex-dependent findings across species should be carefully examined and limited to paradigms that can be applied in the same manner across species.

In the context of cerebellar-dependent learning, evidence in mice shows that estradiol increases the density of parallel fibre to Purkinje cell synapse and induces LTP, which improves memory formation [79] in mice. Experiments using the trace eyeblink conditioning paradigm in mice showed that both sexes reach similar learning scores but females reach significantly higher CR percentage compared to males in the first 5 days of learning [26]. These findings together with our results suggest that female mice exhibit faster learning rates during the first stages of learning. These results in mice are in line with studies performed in humans, where females were observed to produce more CRs than males throughout the duration of the delay eyeblink conditioning paradigm [23]. It should be noted however that, unlike mouse experiments, in human studies, all trials are delivered during a single training day. It would be of interest to investigate whether males reach learning scores comparable to females over several training sessions.

Given that our data and previous reports [26] show faster acquisition of CRs in females of both B6 and B6CBAF1 mouse strains, we postulate that including female mice in future eyeblink studies could reduce the training time to achieve desired scores. This could be advantageous for certain experiments, particularly time-sensitive ones, such as calcium imaging or electrophysiological measurements. In addition, our results show that male and female mice have similar variability regardless of the strain, which indicates that females can be included in studies without tracking the estrous cycle phase.

A common experimental setting in neuroscience involves head-fixing awake mice and placing them on a freely moving wheel. In our experiments, the differences in learning scores between sexes were strongly correlated to the changes in speed on the wheel, which is in line with the previous studies [27]. This suggests that spontaneous locomotion might facilitate cerebellar associative learning and could be predictive of learning scores. Locomotion has long been known to induce theta rhythms in the hippocampus [80–82], which in turn facilitate episodic memory consolidation and hippocampal-dependent learning [83,84], and have been shown to be important for social memory formation [85]. In humans, theta rhythms have been linked to active learning [86] and improved episodic memory recall [87]. It would be of interest to investigate how hippocampal theta rhythms influence cerebellar activity during delay eyeblink conditioning in future research by performing dual cerebellar and hippocampal recordings during eyeblink training.

Another factor that should be taken into consideration when studying sex differences in performance is stress. It is known that stress plays an important role in modulating neural activity in the hippocampus. Corticosterone- among other stress hormones- increases CA1/CA3 firing rates shortly after a stressful period and induces molecular cascades that enhance calcium influx, which disrupts hippocampal function [88]. Similar mechanisms have been described in the cerebellum, where calcium-based excitability in the DCN is altered in animals with higher levels of corticosterone [89].

Regarding locomotion, the evidence on the effects of stress on walking and running behaviours is inconsistent. Some studies using head-fixed male mice showed that these increased wheel running speeds after experiencing different forms of induced stress [90,91]. Another study using male mice but a different form of head fixation, a ‘mobile home cage’ (MHC) container, found that corticosterone levels were generally lower with larger numbers of rotations of the MHC container [92]. However, others found that male mice not habituated to a wheel run less after stress exposure [93]. Studies that include mice from both sexes in their experimental design have mostly been limited to freely moving behaviours and showed that females exhibit increased exploratory behaviour but not running upon stress [94,95]. A recent study that investigated adaptation to head fixation in both sexes showed that, over the 7-day habituation period, both males and females increased the velocity and the time spent running. However, female mice habituated to running forward within the initial 2 days, while male mice took up to 4 days to habituate to running forward [29]. Unfortunately, in our current study, we did not track the movement over the habituation period. Thus, comparisons between our data and the Warner and Padmanabhan results are not possible. Future eyeblink experiments in combination with stress monitoring, such as cortisol measurements and quantification of innate running behaviour in homecages using for example ‘smart housing’ solutions, would help elucidate the contributors to the correlation between locomotion and sex found in our study.

### 3.2. Associative learning networks

Our results show that the expression of c-Fos in the cerebellar cortex, following the short paradigm of delay eyeblink conditioning, is localized to the granule cell layer. This is expected, given that multiple forms of plasticity have been shown within the synapses in this layer. For example, the mossy fibre-granule cell synapse undergoes both LTP and LTD [96], and evidence has shown that granule cell activity adapts over time during eyeblink conditioning [97] and other types of learning [98,99]. In addition, the induction of LTP by theta-burst stimulation in acute cerebellar slices activates a cAMP-responsive element-binding protein cascade which, in turn, activates c-Fos expression [100]. Our results are consistent with these findings and, overall, they provide evidence on how plasticity, at the input level in the cerebellar cortex, can evoke transcriptional processes that contribute to learning consolidation. The strongest correlations between c-Fos expression and CR amplitude within the cerebellum were observed in simplex, lobule VI and crus 1, which is consistent with the ‘eyeblink region’, but expands beyond the small area usually recorded using electrophysiological approaches [12,13]. Strong c-Fos expression in crus 1 supports our previous findings showing the importance of this lobule in eyeblink conditioning [15].

Outside of the cerebellum, we identified several brain areas that could play a role in eyeblink conditioning. At the brainstem level, we found a positive correlation between CR amplitude and c-Fos density in the pons, inferior olive, red nucleus and facial nucleus. Activity in these areas can be expected given that the red and facial nuclei, together with the oculomotor nucleus, execute the blink. Similarly, the inferior olive and the pontine nuclei relay the US and CS information to the cerebellar cortex, respectively. The positive correlation between learning scores and c-Fos expression was significant in the short and the long paradigm, which suggests the importance of these regions in acquisition and consolidation of delay eyeblink conditioning.

Moreover, we found higher c-Fos expression in the visual, motor and somatosensory cortices in mice with higher learning scores at day 5 of learning. Processing in these cortices could facilitate the CS to become more salient and ultimately predict the US. The somatosensory cortex projects to the lateral amygdala which, in turn, projects to the central amygdala, to ultimately contact the pons. The high c-Fos expression found in the amygdala points towards a two-stage conditioning model; where the amygdala would have an initial role with arousal as a salient feature, and a second phase where the cerebellum would take over to form precisely timed CRs [16]. In the motor cortex, higher c-Fos levels in high-performing mice might be due to locomotor activity rather than learning itself. However, as mentioned above, this could play a role in learning either by directly affecting cerebellar input or indirectly as arousal.

The comparison of the short- and long-learning paradigm revealed that the positive correlation between c-Fos density and CR amplitude in brainstem areas remained significant in both paradigms. This suggests that the red nucleus, facial nucleus, inferior olive and pontine nuclei have a similar engagement in early stages as well as in later stages of learning. However, the positive correlations in the cerebellar cortex as well as in the visual, motor, somatosensory cortices and the amygdala were significant in the short paradigm but not significant in the long paradigm. This indicates a distinct involvement of these regions between early and late stages of learning. We have observed that eyeblink scores start to plateau on days 5–6 of training, which marks a transition between early and late learning stages and is consistent with the hypothesized memory transfer from the cerebellar cortex [16,55]. Our results are also congruent with studies which show that the motor cortex is not required for an execution of a learned behaviour once the learning is consolidated [58,101]. Notably, the temporal resolution of the c-Fos staining is very poor and the presence of c-Fos expression alone does not provide us with any information about the activity patterns directly related to the stimuli used in the eyeblink conditioning task [51,52]. Nonetheless, our findings give us a better understanding of the networks underlying eyeblink conditioning and provide candidate brain areas, which can be further studied in the context of associative learning, including methods that measure neuronal activity directly with high temporal resolution (i.e. [102,103]).

## 4. Materials and methods

### 4.1. Animals

C57BL/6J mice were ordered from Charles River (*n* = 27 males; *n* = 27 females), and B6CBAF1 mice from Janvier (*n* = 7 males; *n* = 9 females). Mice were group-housed and kept on a 12 h light-dark cycle with *ad libitum* food and water. All procedures were performed in male and female mice approximately 8–12 weeks of age.

### 4.2. Eyeblink pedestal placement surgery

Mice were anaesthetized with isoflurane and oxygen (4% isoflurane for induction and 2–2.5% for maintenance). Body temperature was monitored during the procedure and maintained at 37°C. Animals were fixed in a stereotaxic device (Model 963, David Kopf Instruments, Tujunga CA, USA). The surgery followed previously described standard procedures for pedestal placement [2,14]. In short, the hair on top of the head was shaved, Betadine and lidocaine were applied on the skin and an incision was done in the scalp to expose the skull. The tissue on top of the skull was removed and the skull was kept dry before applying Optibond prime adhesive (Kerr, Bioggio, Switzerland). A pedestal equipped with a magnet (weight approx. 1 g) was placed on top of the skull with Charisma (Heraeus Kulzer, Armonk NY, USA), which was hardened with UV light. Rimadyl was injected subcutaneously (5 mg per kg). Mice were left under a heating lamp for recovery for at least 3 h. Mice were given 3–4 resting days before starting experiments.

### 4.3. Eyeblink conditioning

Mice were habituated to the set-up (head-fixed to a bar suspended over a cylindrical wheel in a sound and light isolating chamber) for 5 days with increasing exposure (15, 15, 30, 45 and 60 min). Training started after 2 days of rest. Forty-three C57BL/6 mice (*n* = 21 males; *n* = 22 females), and 16 B6CBAF1 mice (*n* = 7 males; *n* = 9 females) were trained using the standard eyeblink protocol [2,12] (figure 1*a*). Ten CS-only trials of 30 ms with an inter-trial interval (ITI) of 10 ± 2 s were presented before the first training session to acquire a baseline measurement. Mice were next trained for 5 (short protocol) or 10 (long protocol) consecutive days. Each session consisted of 20 blocks of 12 trails each (1 US only, 11 paired and 1 CS only) with an ITI of 10 ± 2 s. The CS was a 270 ms blue LED light (approx. 450 nm) placed 7 cm in front of the mouse. The US was a 30 ms corneal air puff co-terminating with the CS. The puffer was controlled by a VHS P/P solenoid valve set at 30 psi (Lohm rate, 4750 Lohms; Internal volume, 30 µl, The Lee Company, Westbrook, USA) and delivered via a 27.5 mm gauge needle at 5 mm from the centre of the left cornea. The puff was tested every day to make sure that the startle response is minimal. Eye traces were monitored at the beginning of experiments and, if necessary, the puff position was adjusted to avoid a startle response. The inter-stimulus interval was 250 ms. Eyelid movements were recorded with a camera (Basler aceA640) at 250 frames s^−1^. Eleven C57BL/6 mice (*n* = 6 males; *n* = 5 females) were trained using a pseudoconditioning protocol. Pseudoconditioning protocol consisted of 20 blocks of 12 trails each (1 puff only, 12 LED only) with an ITI of 10 ± 2 s. The puff and LED stimulus had the same characteristics as in the conditioning protocol. Data were analysed with a custom written MATLAB code as previously described [15,97]. Traces were normalized within each session to the UR max amplitude. The CR detection window was set to 650–730 ms and CRs were only classified as such when the amplitude was equal or higher than 5% of the UR median. The CR percentage was calculated as the number of counted CRs (equal or higher than 5% of the UR median) divided by the total CS trials per session.

### 4.4. Locomotion

An infrared camera (ELP 1080P) (sampling frequency 60 frames s^−1^) was placed in each of the eyeblink boxes and connected to an external computer (independent from the eyeblink system). The cameras were positioned at the right back corner of the chamber on top of a magnet tripod attached to a custom-made metal block which allowed stable fixation. The recording angle was standardized by selecting the same reference in the field of view of each camera. Simultaneous video acquisition from the three cameras was performed in Ipi Recorder software (http://ipisoft.com/download/). Body movement recording was parallel to eyelid recording during the training sessions. The output videos (.avi format) from each mouse and session were approximately 35 min (corresponding to the length of an eyeblink session).

### 4.5. Locomotion analysis

We used DLC to track body parts from videos [56] (electronic supplementary material, figure S1). We extracted 40 frames of four different videos from two males and two females (a total of 160 frames). Next, frames were manually labelled with five body parts (tail base, hip, knee, right back paw and nose). These frames were used for training the pre-trained deep neural network ResNet50 [104,105]. Evaluation of the network was done to confirm a low error in pixels between labelled frames and predictions. Video analysis was done by using the trained network to get the locations of body parts from all mice and sessions [(36 B6 mice short paradigm × 5 days) + (18 B6 mice long paradigm × 10 days) = 360 videos]. DLC output is a matrix with *x* and *y* positions in pixels and the likelihood of this position for each body part. We used this matrix to calculate distance covered and speed per body part with a custom written code (https://github.com/BaduraLab/DLC_analysis). After confirming normal distribution of the spatial coordinates per body part over training sessions, we performed the Grubbs's test for outlier removal to discard possible tracking errors.

### 4.6. Tissue processing

Mice were anaesthetized with 0.2 ml pentobarbital (60 mg ml^−1^) and perfused with 0.9% NaCl followed by 4% paraformaldehyde (PFA). Given the peak time expression of c-Fos [39], animals were perfused 90 min after finishing the last training session. Brains were dissected from the skull and stored in 4% PFA at room temperature (rT) for 1.5 h. They were next changed to a 10% sucrose solution and left overnight at 4°C. Brains were embedded in 12% gelatine and 10% sucrose and left in a solution with 30% sucrose and 4% PFA in PBS at rT for 1.5 h. Next, they were transferred to a 30% sucrose solution in 0.1 PB and kept at 4°C. Whole brains were sliced at 50 µm with a microtome and slices were kept in 0.1 M PB.

### 4.7. Immunostaining and imaging

Sections were incubated in blocking solution (10% NHS, 0.5% Triton in PBS) for an hour at rT. After rinsing, sections were incubated for 48 h at 4°C on a shaker in primary antibody solution with 2% NHS (1:2000 Rabbit anti-c-Fos, ab208942, Abcam; 1:1000 Rat anti-Ctip2, ab18465, Abcam; 1:1000 Rabbit anti-GABAalpha6, G5555, Sigma-Aldrich; 1:1000 Rabbit anti-Cux1) [106]. After rinsing, sections were incubated for 2 h at rT on a shaker with secondary antibody (1:500 Donkey anti-rabbit A594, 711-585-152, Jackson; 1:500 Donkey anti-Rabbit A488, 711-545-152, Jackson; 1:500 Donkey anti-rabbit Cy5, 711-175-152, Jackson; Donkey anti-rat Cy3, 712-165-150, Jackson). Sections were counterstained with DAPI. Finally, sections were rinsed in 0.1 M PB, placed with chroomulin on coverslips and mounted on slide glasses with Mowiol.

Sections were imaged with a Zeiss AxioImager 2 (Carl Zeiss, Jena, Germany) at 10 x. A DsRed filter and an exposure time of 300 ms was used for the Alexa 595 channel (c-Fos). The DAPI channel was scanned at 20 or 30 ms exposure time. Tile scans were taken from whole-brain slices. We processed half the sections obtained from slicing, hence, the distance between tile scan images was 100 µm. High-resolution images were taken with a LSM 700 confocal microscope (Carl Zeiss, Jena, Germany).

### 4.8. Image analysis

We developed an image analysis workflow for brain region identification and quantification of c-Fos positive neurons following eyeblink conditioning (electronic supplementary material, figure S2) (https://github.com/BaduraLab/cell-counting). The workflow combines Fiji and a SHARP-Track, a software written in MATLAB initially developed to localize brain regions traversed by electrode tracks [107] (https://github.com/cortex-lab/allenCCF/tree/master/SHARP-Track). Brain slices were preprocessed (rotating, cropping and scaling) with a custom written macro in Fiji [108]. Next, slices were registered to the Allen Brain Atlas using the SHARP-Track user interface. Segmentation was performed on the registered slices in Fiji. Given the characteristic c-Fos staining pattern in the cerebellar granule layer (figure 3*a*), we used different thresholding algorithms for the cerebellum and for the rest of the brain. Following that step, automated cell counting of c-Fos positive neurons was performed with a custom written macro in Fiji (cerebellum—circularity: 0.5–1, size: 0–20 pixels, rest of the brain—circularity: 0.7–1, size: 0–40 pixels) to get the *x* and *y* coordinates of every detected cell. The output matrix of coordinates was used to create a ROI array per slice in SHARP-Track. This step allows one to one matching between the ROI array and the previously registered slice. Finally, the reference-space locations and brain regions of each neuron were obtained by overlapping the registration array with the ROI array. ROI counts were normalized by brain region surface following the hierarchical structure of the Allen Brain Atlas. The surface of each brain area was calculated per slice and cell density was defined as ROI counts/surface.

### 4.9. Statistics

Statistics were performed in MATLAB and GraphPad Prism 6. Data are reported as mean ± s.d. or s.e.m. Normality was tested and followed for both eyeblink CR amplitudes and for speed of the right back paw. The corresponding statistical test for the *p*-values reported are specified in Results. Time data (training sessions) was analysed using two-way repeated measures ANOVA for sex and session. Data were corrected for multiple comparisons using the Sidak or Tukey tests. Sex effect is reported in Results; session effect is significant in all groups of the short and long paradigm (indicating learning over time) and interaction is reported if significant. For Kendall's correlation on c-Fos data, we report tau and *p*-values. This correlation method was chosen because it does not make assumptions on the data distribution (unlike Pearson's correlation) [109]. For the analysis of c-Fos density in distinct brain areas, a two-way ANOVA with Tukey's multiple comparisons test was used for the short paradigm data. Given the low number of non-learners in the long paradigm, data weres analysed with a Kruskal–Wallis and Dunn's multiple comparisons test.

## Data Availability

The code described in the paper is available online at https://github.com/BaduraLab/. The supporting data can be found online at https://github.com/BaduraLab/Eyeblink. The data are provided in the electronic supplementary material [110].
